# Retrotransposition Events Shape the Evolution of the Ataxin-3 Gene Family in Primates

**DOI:** 10.1093/gbe/evag047

**Published:** 2026-03-12

**Authors:** Daniela Felício, Inês M Martins, Andreia Pinto, Jorge Sequeiros, António Amorim, Alexandra M Lopes, Susana Seixas, Sandra Martins

**Affiliations:** i3S - Instituto de Investigação e Inovação em Saúde, University of Porto, Porto, Portugal; Institute of Molecular Pathology and Immunology of the University of Porto (IPATIMUP), University of Porto, Porto, Portugal; Instituto Ciências Biomédicas Abel Salazar (ICBAS), University of Porto, Porto, Portugal; Institute of Molecular Pathology and Immunology of the University of Porto (IPATIMUP), University of Porto, Porto, Portugal; Institute of Molecular Pathology and Immunology of the University of Porto (IPATIMUP), University of Porto, Porto, Portugal; i3S - Instituto de Investigação e Inovação em Saúde, University of Porto, Porto, Portugal; Instituto Ciências Biomédicas Abel Salazar (ICBAS), University of Porto, Porto, Portugal; i3S - Instituto de Investigação e Inovação em Saúde, University of Porto, Porto, Portugal; Institute of Molecular Pathology and Immunology of the University of Porto (IPATIMUP), University of Porto, Porto, Portugal; Department of Biology, Faculty of Sciences, University of Porto, Porto, Portugal; i3S - Instituto de Investigação e Inovação em Saúde, University of Porto, Porto, Portugal; Institute of Molecular Pathology and Immunology of the University of Porto (IPATIMUP), University of Porto, Porto, Portugal; CGPP-IBMC – Centro de Genética Preditiva e Preventiva, Instituto de Biologia Molecular e Celular, Universidade do Porto, Portugal; i3S - Instituto de Investigação e Inovação em Saúde, University of Porto, Porto, Portugal; Institute of Molecular Pathology and Immunology of the University of Porto (IPATIMUP), University of Porto, Porto, Portugal; i3S - Instituto de Investigação e Inovação em Saúde, University of Porto, Porto, Portugal; Institute of Molecular Pathology and Immunology of the University of Porto (IPATIMUP), University of Porto, Porto, Portugal

**Keywords:** paralog genes, retrotransposition, repeat expansion diseases, Machado–Joseph disease/spinocerebellar ataxia type 3, ataxin-3

## Abstract

Evolutionary studies of disease-associated genes provide crucial insights into pathological mechanisms and potential therapeutic targets. Polyglutamine spinocerebellar ataxias (SCAs) are human neurodegenerative diseases caused by toxic expanded CAG repeats. Studies on SCA1 have shown that a paralog of the causing-gene can partially rescue protein function and alleviate the neuropathology. The most common SCA, Machado–Joseph disease (MJD/SCA3), caused by mutated ataxin-3 gene (*ATXN3*), has no treatment currently available. Its paralog ataxin-3 like (*ATXN3L*) remains largely unexplored. Here, we identify three new retrotransposition events of *ATXN3*: *ATXN3L0* in Euarchontoglires, *ATXN3L2* in Simiformes, and *ATXN3L3* in Cercopithecidae, in addition to *ATXN3L* (herein called *ATXN3L1*) originated in Haplorrhini. *ATXN3* and *ATXN3L1* are both under purifying selection throughout primate evolution, maintaining about 70% of amino acid identity. Also, the high conservation of ATXN3L1 Josephin domain hints at functional redundancy with the parental disease-associated *ATXN3*. *ATXN3L2* presents a remarkable nucleotide similarity to *ATXN3* (79%) in an interrupted reading frame, which may produce a regulatory RNA. Conversely, *ATXN3L0* is likely a non-functional retrocopy and *ATXN3L3* is absent in humans with no relevance for the disease. The comparison of (CAG)_n_ interruption patterns of the different paralogs in several primates elucidates the process leading to the currently observed pure long tracts in human *ATXN3*, responsible for disease when expanded. This study intends to pioneer the identification of new paralogs of SCA-associated genes and the use of phylogenetic analyses to explore their potential role for targeted therapies.

Significance Polyglutamine spinocerebellar ataxias are human progressive brain diseases caused by the abnormal expansion of repetitive DNA sequences in particular genes. These expansions lead to the production of faulty proteins that accumulate in nerve cells, ultimately causing their dysfunction and degeneration over time. Previously, other researchers have found that similar counterparts of these disease-associated genes in the human genome (gene copies) are able to compensate for the loss of normal protein function, offering potential therapeutic targets.

In this study, we discovered new copies of the gene responsible for the most frequent of these dominant ataxias worldwide. Our findings highlight an approach that can be followed to identify similar gene copies linked to other repeat-based disorders and select the most promising therapeutic candidates.

## Introduction

Gene duplication contributes to the evolution of species by the acquisition of new genetic material for mutation, genetic drift, and selection to act upon (Innan and Kondrashov 2010). Traditionally, duplicate genes are reported to originate through a DNA-based mechanism, often via unequal crossing over (from segmental or tandem duplications), thus, maintaining a similar organization and physical proximity to the parental gene (Kaessmann 2010). However, RNA-based duplication, termed retrotransposition or retroduplication, represents another source of gene duplication, thereby generating intronless copies (retrocopies) that can be located elsewhere in the genome (Kaessmann et al. 2009). In this instance, the processed mRNA of the parental gene undergoes reverse transcription and random incorporation by the long interspersed nuclear elements-1 (LINE1 or L1) machinery. Given that retrocopies generally lack transcriptional elements, they were thought to be non-functional copies designated as retropseudogenes or processed pseudogenes. Therefore, they were long believed to be free from selective pressures, accumulating diverse loss of function mutations such as premature stop codons and frameshift mutations (Kaessmann et al. 2009). Notwithstanding, there are functional retrogenes with retained or acquired promoter elements, which potentiate their transcription (Casola and Betrán 2017).

The identification of gene paralogs (originated through DNA- or RNA-based duplications) is not only crucial to obtain a complete picture of the evolution of organisms but also to identify potential pathological buffering mechanisms that might be applied as therapies when the parental gene is associated to disease (Felício et al. 2023). Spinocerebellar ataxias (SCAs) are a heterogeneous group of neurodegenerative disorders characterized by progressive cerebellar ataxia, resulting in lack of muscle control and coordination. In this group of autosomal dominant disorders, seven are classified as polyglutamine ataxias since they result from the elongation of a polyglutamine (polyQ) tract encoded by an expanded CAG repeat (Klockgether et al. 2019). Those are SCA1, SCA2, MJD (Machado–Joseph disease)/SCA3, SCA6, SCA7, SCA17, and dentatorubral-pallidoluysian atrophy (DRPLA). More recently, researchers found a new CAG repeat expansion in the coding region of *THAP11* associated with spinocerebellar ataxia in two Chinese families, which may suggest that the group of polyQ SCAs will expand in the future (Tan et al. 2023). In these disorders, elongated polyQ stretches promote protein misfolding and aggregation, which inhibit the native function and/or lead to abnormal protein interactions (Klockgether et al. 2019).

Interestingly, studies in SCA1 demonstrated that a highly conserved DNA-based copy of the human disease-associated *ATXN1* gene (called ataxin-1 like; *ATXN1L*; Mizutani et al. 2005) was able to partially compensate the loss of wild-type *ATXN1* function, modulating the cytotoxicity of expanded *ATXN1* and suppressing SCA1 neuropathology in mice (Bowman et al. 2007; Crespo-Barreto et al. 2010; Carrell et al. 2022). Indeed, ATXN1 and ATXN1L share a conserved AXH (ataxin-1 and HMG-box protein 1) domain (66% identity), essential for their interaction with each other and with the transcriptional corepressor Capicua (Mizutani et al. 2005; Kim et al. 2013). In short, the overexpression of *ATXN1L* enabled the paralog to compete with the mutant ATXN1 for the interaction with Capicua, partially rescuing the transcriptional complex, a known mechanism that drives cerebellar pathology in SCA1 (Lam et al. 2006; Bowman et al. 2007).

The most common dominant ataxia worldwide is MJD/SCA3 (Sequeiros et al. 2012; Martins and Sequeiros 2018). Similar to other polyQ disorders, its causing gene, ataxin-3 (*ATXN3*, 14q32.12) contains a CAG repeat, which normally ranges from 12 to 44 CAGs. Conversely, affected individuals usually present expanded alleles surpassing the 61 repeats, with the expansion size partially explaining the variability in the age-at-onset, which can vary from infancy to later in adulthood (average onset at 40 years old) (Conceição and Manuela 2011). Thus, modifiers of disease phenotype must play an important role in MJD. The native function of wild-type ATXN3 has been associated with proteostasis, through its deubiquitinase activity (Burnett et al. 2003; Burnett and Pittman 2005), and with transcriptional regulation activities by its binding to DNA and several transcription-related factors (Li et al. 2002; Evert et al. 2006).

*ATXN3* belongs to an old gene lineage found in non-bilateria organisms, suggesting that this gene performs essential functions (Sousa e Silva et al. 2023). A human paralog of *ATXN3* is already known to exist in primates (*ATXN3L;* Xp22.2) (Scheel et al. 2003; Vlasschaert et al. 2017; Sousa e Silva et al. 2023), but very few studies have investigated *ATXN3L* from an evolutionary standpoint; thus, *ATXN3L* still lacks a full taxonomic annotation, the same way that other paralogs may remain unknown. No ATXN3L protein has been detected so far, but an in vitro experiment demonstrated that, if translated, ATXN3L may present higher deubiquitinase activity than the parental ATXN3. Interestingly, the replacement of three ATXN3 residues (S12F, R59L, and T60A) was found to almost reach the level of ATXN3L activity (Weeks et al. 2011). Since both proteins show distinct ubiquitin recognition sites (Weeks et al. 2011), it would be interesting to explore evolutionary constraints of ATXN3/ATXN3L residues and their biological relevance.

To this end, we aimed to reconstruct the evolutionary history of *ATXN3* family/paralogs and to conceptually explore their roles as MJD modifiers. To this end, we conducted an exhaustive analysis of *ATXN3* homologs by retrieving the sequences of 33 primate species and other close mammals from genomic databases. Our analyses allowed us to identify four main retrocopies of *ATXN3* in primates, originated at different time points in mammalian evolution. To explore the relevance of *ATXN3* copies, we examined sequence diversity, gene structure, and selective pressures acting on *ATXN3* paralogs during evolution. Our results suggest that *ATXN3L* (herein called *ATXN3L1*) and the newly identified *ATXN3L2* are the most promising paralogs to proceed with functional characterization and explore their role and interaction with the parental gene. Finally, our *in-silico* approach aims to pioneer the study of other SCA-associated paralogs and their potential to affect and/or partially maintain the function of the respective parental genes.

## Results

### *ATXN3* Retrotransposition Events in Primates

**Fig. 1.**
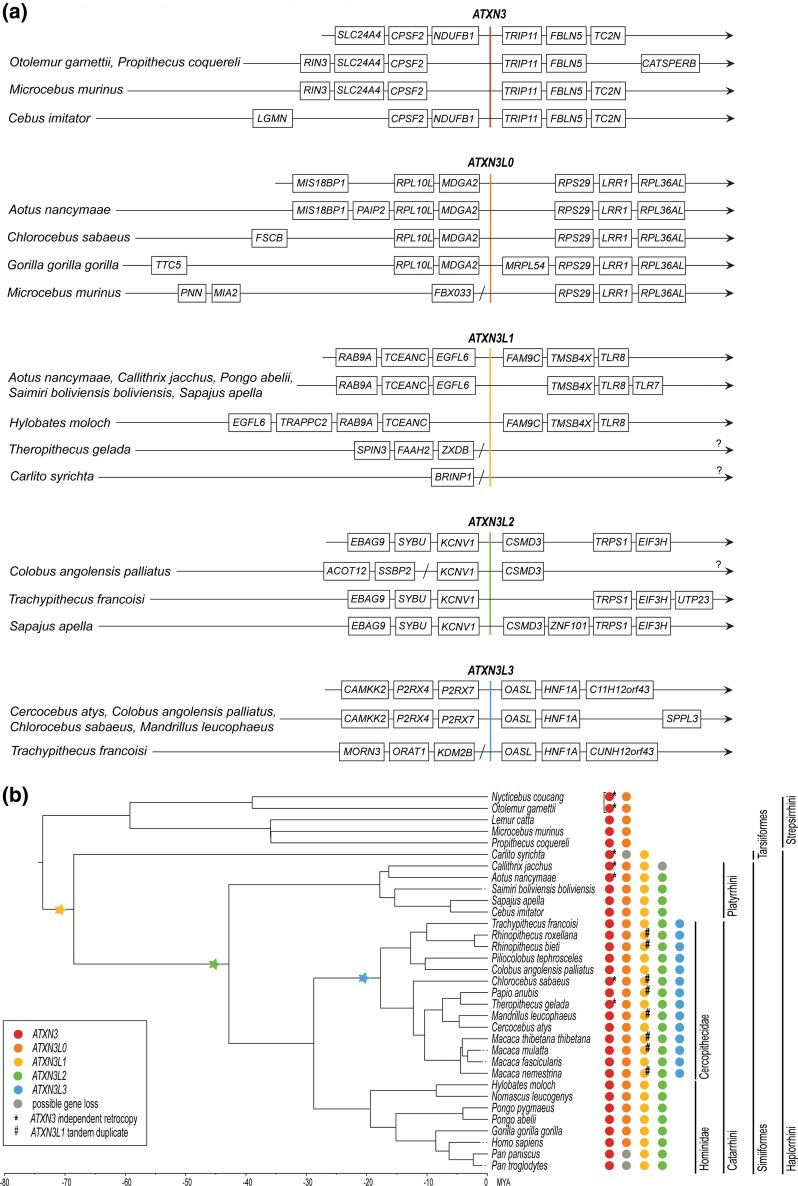
Reconstruction of *ATXN3* gene family evolution in primates. a) Local synteny information of the different *ATXN3* paralogs in primate species, with the top rows showing synteny observed in each retrocopy for most species followed by exceptions. Interrogation points (?) represent lack of annotation in NCBI assemblies. Slashes (/) represent complete loss of synteny in the respective direction. b) Primate phylogenetic tree showing the origin of the different *ATXN3* copies (obtained from TimeTree, https://timetree.org/). The presence of *ATXN3* paralogs is marked in each species with colored circles. A square bracket ([) indicates two copies from a common ancestor. The stars represent the time at which the retrotranspositions seem to have occurred. MYA, million years ago.

Upon the analysis of the sequence similarity between the coding sequence of the human *ATXN3* (*hsaATXN3*) and 33 primate genomes, we identified four common retrocopies named *ATXN3L0* to *ATXN3L3* (Fig. 1, Table S1) through the analysis of synteny (genes flanking each retrocopy; Fig. 1a) and sequence identity between orthologs and paralogs (Table S2a). Next, we estimated the timing of each retrotransposition based on their most recent common ancestor in the primate or mammalian species tree (Fig. 1): *ATXN3L0* (81.3 to 91.0 MYA)*, ATXN3L1* (formerly known as *ATXN3L*; 61.6 to 71.1 MYA), *ATXN3L2* (40.0 to 44.2 MYA), and *ATXN3L3* (15.50 to 19.80 MYA), next described in detail.

*ATXN3L0* is the only copy present in most primates, with possible gene loss events occurred in chimpanzees (*Pan troglodytes* and *Pan paniscus*) and tarsiers (*Carlito syrichta*; Table 1, Fig. 1b). Several premature stop codons observed along the *ATXN3L0* phylogeny indicated that this is likely an ancient processed pseudogene (located 42 Mb away from the parental gene in humans; LOC100418768; 14q21.3; Table S1). Among non-primate mammals, we identified sequences likely to be homologous to *ATXN3L0*, namely in the closest evolutionary relatives to primates (flying lemurs and treeshrews) and among rodents (in the Sciuridae family).

**Table 1:** Four main *ATXN3* paralogs identified in primate species (NCBI database)

Species name	*ATXN3L0*	*ATXN3L1*	*ATXN3L2*	*ATXN3L3*
*Homo sapiens*	LOC100418768	*ATXN3L*	LOC100132280	*─*
*Pan paniscus*	─	*ATXN3L*	LOC100968165	*─*
*Pan troglodytes*	**─**	LOC129138637	LOC100613414	*─*
*Gorilla gorilla gorilla*	NA	*ATXN3L*	LOC101124755	*─*
*Pongo abelii*	NA	*ATXN3L*	LOC100455664	*─*
*Pongo pygmaeus*	NA	*ATXN3L*	LOC129042124	*─*
*Hylobates moloch*	NA	*ATXN3L*	LOC116813850	*─*
*Nomascus leucogenys*	NA	*ATXN3L*	LOC100601102	*─*
*Macaca mulatta*	NA	LOC711119	LOC700434	LOC699321
		LOC114674839^a^		
*Macaca fascicularis*	NA	*ATXN3L*	LOC102117545	LOC102119590
*Macaca thibetana thibetana*	NA	*ATXN3L*	LOC126960647	LOC126930683
		LOC126945336^a^		
*Macaca nemestrina*	NA	*ATXN3L*	LOC105476127	LOC105494606
		LOC105478274^a^		
*Mandrillus leucophaeus*	NA	LOC105550045	LOC105555234	LOC105527940
		LOC105550026^a^		
*Cercocebus atys*	NA	*ATXN3L*	LOC105584571	LOC105590961/LOC105590962
*Papio anubis*	NA	*ATXN3L*	LOC110743999	LOC101017058
		LOC101021868^a^		
*Theropithecus gelada*	NA	*ATXN3L*	LOC112630196	LOC112635516
*Chlorocebus sabaeus*	NA	LOC103231613	LOC103237716	LOC119623360/LOC103239276
		LOC119619931^a^		
*Colobus angolensis palliatus*	NA	ATXN3L	LOC105506288	LOC105516342/LOC105516344
*Piliocolobus tephrosceles*	NA	*ATXN3L*	LOC111549645	LOC111537833
*Rhinopithecus bieti*	NA	LOC108523901/LOC108544961	LOC108541821	LOC108529641
		LOC108523899^a^	…	…
*Rhinopithecus roxellana*	NA	*ATXN3L*	LOC104664224	LOC115900089
		LOC104669352^a^	…	…
*Trachypithecus francoisi*	NA	*ATXN3L*	LOC117079069	LOC117089275
*Cebus imitator*	NA	*ATXN3L*	LOC108291766	*─*
*Sapajus apella*	NA	*ATXN3L*	LOC116529997	*─*
*Saimiri boliviensis boliviensis*	NA	*ATXN3L*	LOC101048375	*─*
*Aotus nancymaae*	NA	*ATXN3L*	LOC105710782	*─*
*Callithrix jacchus*	NA	*ATXN3L*	*─*	*─*
*Carlito syrichta*	─	LOC103268037	*─*	*─*
*Microcebus murinus*	NA	*─*	*─*	*─*
*Propithecus coquereli*	LOC105813271	*─*	*─*	*─*
*Lemur catta*	NA	*─*	*─*	*─*
*Otolemur garnettii*	NA	*─*	*─*	*─*
*Nycticebus coucang*	NA	*─*	*─*	*─*

In some species, different contigs (separated by a slash) were found to represent the same gene; NA, not annotated; ─, not found.

^a^Tandem duplicates of *ATXN3L1*.

*ATXN3L1* (Xp22.2 in humans) displayed 69% to 80% sequence identity among all Simiiformes where it was identified (Fig. 1b, Table 1 and Table S2a; exceptions in Fig. S1). Tarsiers were considered to have an *ATXN3L1* copy (despite lacking synteny evidence, Fig. 1a) given that *Carlito syrichta* sequence showed 72% identity to *hsaATXN3* (similar to values observed for the remaining orthologs; Fig. 1, Table S2a). This highlights the importance of integrative analyses since a different synteny from the consensus does not invalidate gene paralogy (as also shown by the annotated *ATXN3L* from *Theropithecus* gelada; Fig. 1a). Notably, in *Carlito syrichta*, we found a second copy of *ATXN3L1* (LOC103249688), sharing 95% sequence similarity, compatible with a recent species-specific retrotransposition event (Table S2a). Finally, other *ATXN3L1* tandem duplication events seem to have occurred in a Cercopithecidae ancestor (Fig. 1b). Duplicates are located about 17 to 22 Kb from *ATXN3L1* and conserve intact reading frames, most of them displaying a very high sequence similarity (99% to 100%) with the respective *ATXN3L1* source gene. While analyzing local synteny, we also noted the lack of consensus in current annotation regarding the location of *ATXN3L1* and its tandem duplicate between species (Table S1).

We identified *ATXN3L2* (LOC100132280; 8q23.2 in humans) in most Simiiformes (Table 1, Fig. 1b) exhibiting an average nucleotide identity of 79% with the respective parental *ATXN3* (Table S2a). Nevertheless, in contrast to *ATXN3L1, ATXN3L2* presents premature stop codons in all analyzed species.

*ATXN3L3* was identified only in Cercopithecidae, displaying high similarity with the respective *ATXN3* paralog (87% to 97% nucleotide identity; Fig. 1b, Table S2a), but different open reading frames are predicted among Old-World monkeys (primates that belong to the family Cercopithecidae). This younger retrotransposed gene is absent in Homoinidea species.

Finally, we detected additional events of *ATXN3* retrotransposition in different primates (*Theropithecus gelada*, *Chlorocebus sabaeus*, *Aotus nancymaae*, *Callithrix jacchus*, and *Carlito syrichta*) and in the common ancestor of *Nycticebus coucang* and *Otolemur garnettii* (Lorisoidea; 34.5 to 40.7 MYA), all classified by us as independent retrocopies (Fig. 1b, Table S1).

### (CAG)_n_ Region of *ATXN3* Paralogs

To address possible mechanisms leading to human-specific (CAG)_n_ expansion in *ATXN3* we have analyzed the repetitive tract across the identified *ATXN3* orthologs and paralogs. Briefly, we aligned the repeat regions of *ATXN3*, *ATXN3L1, ATXN3L2*, and *ATXN3L3* for all primate sequences available, taking into consideration that all may present some level of polymorphism. We excluded *ATXN3L0* from this analysis because no (CAG)_n_ tract was detected in this retrocopy.

The *ATXN3* repeat, which in humans is characterized by the (CAG)_2_ CAA AAG CAG CAA (CAG)_n_ motif, is apparently conserved in half of the analyzed primate species, with only five species showing pure glutamine tracts (CAG/CAA codons) with variable lengths (Table 2).

**Table 2:** Configuration of the (CAG)_n_ tract in primate genome reference sequences of *ATXN3* and its retrocopies

Species	*ATXN3*	*ATXN3L1*	*ATXN3L2*	*ATXN3L3*
*Homo sapiens*	(CAG)_2_ CAA AAG CAG CAA (CAG)_8_	CAT (CAG)_2_ GAA CAG AAG (CAG)_2_ (CAA)_2_ CAG	(CAG)_3_ (CGG CAG)_8_	─
*Pan paniscus*	(CAG)_2_ CAA AAG CAG CAA (CAG)_14_	CAT (CAG)_2_ GAA CAG AAG (CAG)_2_ (CAA)_2_ CAG	CAG (CGG CAG)_7_ GGG CAG	─
*Pan troglodytes*	(CAG)_2_ CAA AAG CAG CAA (CAG)_12_	CAT (CAG)_2_ GAA CAG AAG (CAG)_2_ (CAA)_2_ CAG	CAG (CGG CAG)_8_ GGG CAG	─
*Gorilla gorilla gorilla*	(CAG)_2_ CAA AAG (CAG)_4_	CAT (CAG)_2_ GAA CAG AAG (CAG)_2_ (CAA)_2_ CAG	(CAG)_10_ GAG	─
*Pongo abelii*	(CAG)_2_ CAA AAG CAG CAA (CAG)_2_ CCG CAA (CAG)_2_ CAA (CAG)_2_ CCG CAA (CAG)_7_	CAT (CAG)_2_ GAA CAG AAG CTG CAG (CAA)_2_ CAG	(CAG)_6_	─
*Pongo pygmaeus*	(CAG)_2_ CAA AAG CAG CAA (CAG)_2_ CCG CAA (CAG)_2_ CAA (CAG)_2_ CCG CAA (CAG)_7_	CAT (CAG)_2_ GAA CAG AAG CTG CAG (CAA)_2_ CAG	(CAG)_6_	─
*Hylobates moloch*	(CAG)_2_ CAA AAG CAG CAA (CAG)_7_	CAT (CAG)_2_ GAA CAG AAG (CAG)_2_ (CAA)_2_ CAG	(CAG)_5_	─
*Nomascus leucogenys*	(CAG)_3_ CAA (CAG)_8_	CAT (CAG)_2_ GAA CAG AAG (CAG)_2_ (CAA)_2_ CAG	(CAG)_8_	─
*Macaca mulatta* ^b^	(CAG)_2_ CAA (CAG)_2_ AAG (CAG)_7_	CAT (CAG)_2_ GAA CAG AAG (CAG)_2_ (CAA)_2_ CAG	(CAG)_6_ CAC CAG	(CAG)_2_ CAA AAG CAG CAA (CAG)_2_ AAG (CAG)_5_ GAG (CAG)_2_
*Macaca fascicularis*	(CAG)_2_ CAA (CAG)_2_ AAG (CAG)_7_	CAT (CAG)_2_ GAA CAG AAG (CAG)_2_ (CAA)_2_ CAG	(CAG)_6_ CAC CAG	(CAG)_2_ CAA AAG CAG CAA (CAG)_2_ AAG (CAG)_5_ GAG (CAG)_2_
*Macaca thibetana thibetana* ^b^	(CAG)_2_ CAA (CAG)_2_ AAG (CAG)_11_	CAT (CAG)_2_ GAA CAG AAG (CAG)_2_ (CAA)_2_ CAG	(CAG)_6_ CAC CAG	(CAG)_2_ CAA AAG CAG CAA (CAG)_2_ AAG (CAG)_5_ GAG (CAG)_2_
*Macaca nemestrina* ^b^	(CAG)_2_ CAA (CAG)_2_ AAG (CAG)_10_	CAT (CAG)_2_ GAA CAG AAG (CAG)_2_ (CAA)_2_ CAG	CAG CAA (CAG)_4_ CAC CAG	(CAG)_2_ CAA AAG (CAA)_2_ (CAG)_2_ AAG (CAG)_5_ GAG (CAG)_2_
*Mandrillus leucophaeus* ^b^	(CAG)_2_ CAA AAG CAG CAA (CAG)_2_ AAG (CAG)_10_	CAT (CAG)_2_ GAA CAG AAG (CAG)_2_ (CAA)_2_ CAG	(CAG)_6_ CAC CAG	(CAG)_2_ CAA AAG (CAG)_5_ GAG (CAG)_2_
*Cercocebus atys*	(CAG)_2_ CAA AAG CAG CAA (CAG)_2_ AAG (CAG)_7_	CAT (CAG)_2_ GAA CAG AAG (CAG)_2_ (CAA)_2_ CAG	CAG CAT (CAG)_4_ CAC CAG	(CAG)_2_ CAA AAG (CAG)_5_ GAG (CAG)_2_
*Papio anubis* ^b^	(CAG)_2_ CAA AAG CAG CAA (CAG)_2_ AAG (CAG)_7_	CAT (CAG)_2_ GAA CAG AAG (CAG)_2_ (CAA)_2_ CAG	(CAG)_7_ CAC CAG	(CAG)_2_ CAA AAG (CAG)_5_ GAG (CAG)_2_
*Theropithecus gelada* ^a^	(CAG)_2_ CAA AAG CAG CAA (CAG)_2_ AAG (CAG)_7_	CAT (CAG)_2_ GAA CAG AAG (CAG)_2_ (CAA)_2_ CAG	CAG CAT (CAG)_5_ CAC CAG	(CAG)_2_ CAA AAG (CAG)_5_ GAG (CAG)_2_
*Chlorocebus sabaeus* ^a b^	(CAG)_2_ CAA AAG CAG CAA (CAG)_8_ AAG (CAG)_3_ AAG (CAG)_6_	CAT CGG CAG GAA CAG AAG (CAG)_2_ (CAA)_2_ CAG	(CAG)_5_ (CAC)_3_ CAG	(CAG)_2_ CAA AAG CAG CAA (CAG)_2_ AAG (CAG)_5_ GAG (CAG)_2_
*Colobus angolensis palliatus*	Absent	CAT (CAG)_2_ GAA CAG AAG (CAG)_4_ CAA CAG	(CAG)_6_ CAC CAG	(CAG)_2_ CAA AAG CAG CAA (CAG)_6_ GAG (CAG)_2_
*Piliocolobus tephrosceles*	(CAG)_2_ CAA AAG (CAG)_4_ AAG (CAG)_5_	CAT (CAG)_2_ GAA CAG AAG (CAG)_2_ (CAA)_2_ CAG	(CAG)_3_ CAT (CAG)_2_ CAC CAG	(CAG)_2_ CAA AAG CAG CAA (CAG)_2_ AAG (CAG)_8_ GAG (CAG)_2_
*Rhinopithecus bieti* ^b^	(CAG)_2_ CAA AAG CAG CAA (CAG)_2_ AAG (CAG)_5_	CAT (CAG)_2_ GAA CAG AAG (CAG)_2_ (CAA)_2_ CAG	(CAG)_8_ CAC CAG	GAG CAG CAA AAG CAG CAA (CAG)_2_ AAG (CAG)_3_ AAG (CAG)_2_ CAA (CAG)_3_ GAG (CAG)_2_
*Rhinopithecus roxellana* ^b^	(CAG)_2_ CAA AAG CAG CAA (CAG)_2_ AAG (CAG)_5_	CAT (CAG)_2_ GAA CAG AAG (CAG)_2_ (CAA)_2_ CAG	(CAG)_9_ CAC CAG	GAG CAG CAA AAG CAG CAA (CAG)_2_ AAG (CAG)_3_ AAG (CAG)_6_ GAG (CAG)_2_
*Trachypithecus francoisi*	(CAG)_2_ CAA AAG CAG CAA (CAG)_2_ AAG (CAG)_5_	CAT (CAG)_2_ GAA CAG AAG (CAG)_2_ (CAA)_2_ CAG	(CAG)_7_ CAC CAG	GAG CAG CAA AAG CAG CAA (CAG)_5_ GAG (CAG)_7_
*Cebus imitator*	CAG CAA (CAG)_4_ (CAA)_2_ CAG CAA (CAG)_8_	GAT GAG CAA GAA CAA (CAG)_2_ AGG AAA CAA AAG	GAG (CAG)_3_ CAA	─
*Sapajus apella*	CAG CAA (CAG)_4_ (CAA)_2_ (CAG)_8_	GAT GAG CAA GAA CAA (CAG)_2_ AGG AAA CAA AAG	GAG (CAG)_3_ CAA	─
*Saimiri boliviensis boliviensis*	CAG CAA (CAG)_5_ (CAA)_5_ CAG CAA (CAG)_12_	GAC GAG CAA GAA CAA (CAG)_2_ AGG AAA CAA AAG	GAG (CAG)_4_ CAA	─
*Aotus nancymaae* ^a^	Absent	CAT GAG CAA GAA CAA (CAG)_2_ AGG AAA CAA AAG	GAG (CAG)_3_ CAA	─
*Callithrix jacchus* ^a^	CAG CAA (CAG)_5_ CAA (CAG)_3_ GAG (CAG)_3_ GAG (CAG)_3_	CAT GAG CAA GAA CAA (CAG)_2_ AGG AAA CAA AAG	─	─
*Carlito syrichta* ^a^	(CAA)_2_ (CAG)_2_	CAG TGG CGG CAG (CAC)_2_ CAG GAC (CAC)_4_ TGC CAA CAG	─	─
*Microcebus murinus*	(CAG)_17_ AAG	─	─	─
*Propithecus coquereli*	Absent	─	─	─
*Lemur catta*	(CAG)_8_ CGG (CAG)_3_	─	─	─
*Otolemur garnettii* ^a^	(CAG)_5_ CAA (CAG)_5_ AAG CGG GAG	─	─	─
*Nycticebus coucang* ^a^	CAG CTG (CAG)_3_ AAG (CAG)_5_ AAG CAG CGG GAG	─	─	─

─, not found.

^a^ *ATXN3* recent species-specific retrocopies: *Theropithecus gelada* LOC112629965 - (CAG)_2_ CAA AAG CAG CAA (CAG)_2_ AAG (CAG)_8_, *Chlorocebus sabaeus* LOC119623504: CAT CGG CAG GAA CAG AAG (CAG)_2_ (CAA)_2_ CAG, *Aotus nancymaae* LOC105706409 - CAG CAA (CAG)_12_, *Callithrix jacchus* LOC100388307 - CAG CAA (CAG)_3_, *Callithrix jacchus* LOC100393745 - (CAG)_7_ CAA (CAG)_5_, *Carlito syrichta* LOC103249688 - CAG CAA (CAG)_2_, *Otolemur garnettii* unknown copy: absent, *Nycticebus coucang* LOC128584333 - CAG CAA (CAG)_2_ CAA.

^b^Tandem duplicates of *ATXN3L1*: *Macaca nemestrina* LOC105478274 - AAG CTA -AT ATT (CAG)_2_ GAA CAA (AGC)_3_ (AAC)_2_ -AG, *Chlorocebus sabaeus* LOC103231613 - TTG CTC GGC AGG GAA CAG AAG (CAG)_2_ CAA CAG CGC -AG. *Macaca mulatta* LOC711119, *Macaca thibetana thibetana* LOC126945336, *Mandrillus leucophaeus* LOC105550026, *Papio anubis* LOC101021868, *Rhinopithecus bieti* LOC108523899 and *Rhinopithecus roxellana* LOC104669352 tandem duplicates have the same (CAG)_n_ configuration as their counterpart *ATXN3L1* represented in the table.

*ATXN3L1* displays a shorter and more interrupted repeat in comparison to *ATXN3* (Table 2). Notably, we found the *ATXN3L1* repeat motif to be conserved in most Catarrhini (CAT (CAG)_2_ GAA CAG AAG (CAG)_2_ (CAA)_2_ CAG), whereas New-World monkeys (from the Platyrrhini parvorder) and *Carlito syrichta* presented more complex repetitive regions.

*ATXN3L2* presents a polymorphic hexanucleotide (CGG CAG)_n_ flanked by CAG and GGG CAG triplets in humans and chimpanzees (Table 2), while the remaining species have mostly pure or simpler CAG repeats.

The *ATXN3L3* repetitive region is very similar to the one in *ATXN3*, presenting the same initial pattern (CAG)_2_ CAA AAG CAG/CAA except for Asian colobine species (*Rhinopithecus* and *Trachypithecus*; Table 2), which display more interrupted sequences.

### Characterization of Retrocopies in the Human Genome

**Fig. 2.**
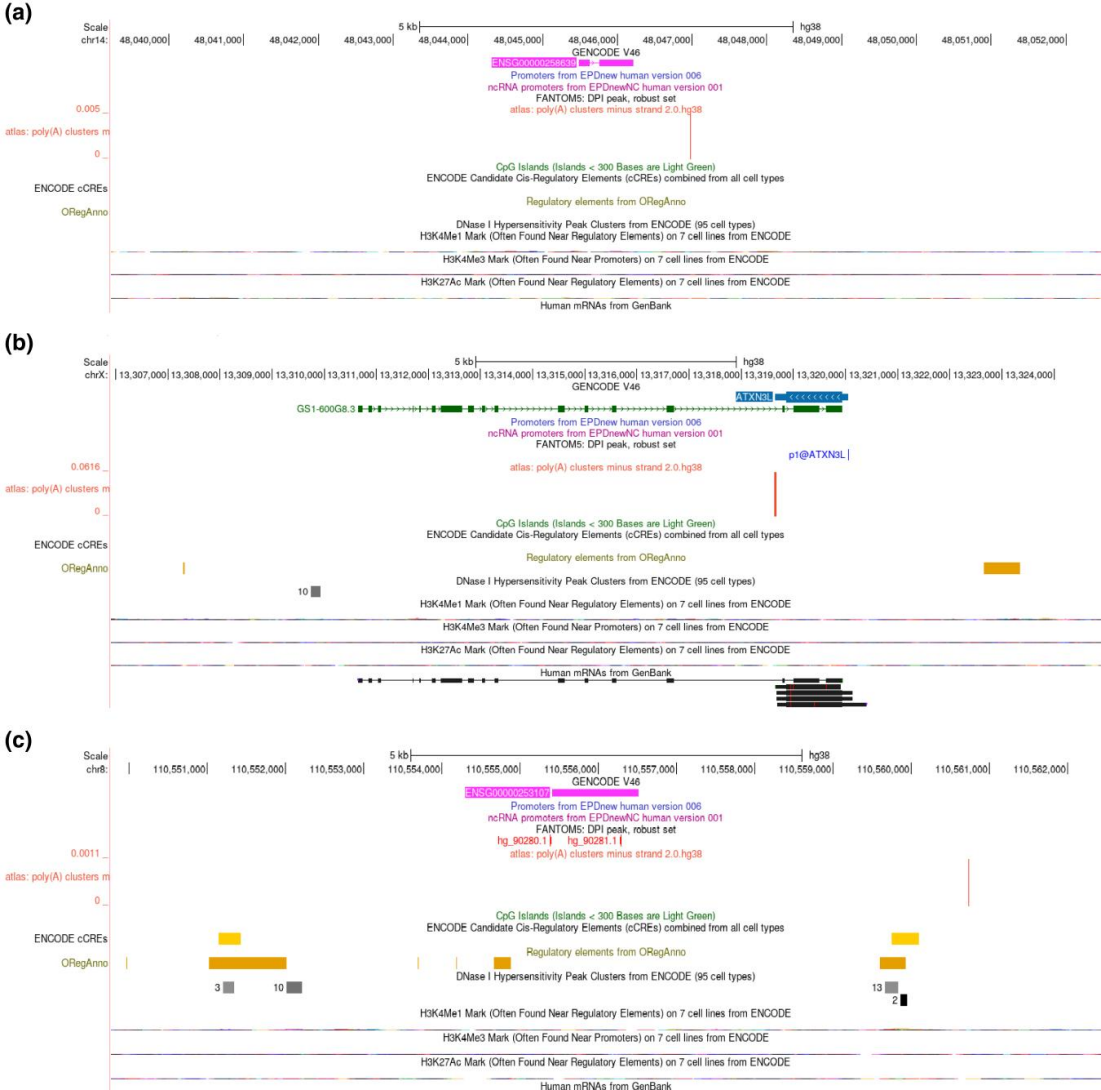
Genomic characterization of *ATXN3* human paralogs. UCSC Genome Browser view displaying summarized regulatory and chromatin-related features (ENCODE, ORegAnno), transcription start site (TSS; FANTOM5), and poly(A) sites (custom track from PolyASite v.2.0) for a) *ATXN3L0*, b) *ATXN3L1*, and c) *ATXN3L2*.

In humans, whereas *ATXN3L0* and *ATXN3L2* are in different intergenic regions (chr14 and chr8, respectively; Fig. 2a and 2c), *ATXN3L1* shares genomic coordinates with an antisense long non-coding RNA (lncRNA) gene in chromosome X (Fig. 2b).

As anticipated, *ATXN3L0* (ENSG00000258639) consists of two exons without a putative transcription start site (TSS) and a polyadenylation (polyA) signal, which indicates it is unlikely to be transcribed (Fig. 2a).

*ATXN3L1* (ENSG00000123594) is a single exon gene that overlaps the last three exons of GS1-600G8.3 (ENSG00000231216.1), an unknown antisense gene of 17 exons that produces a lncRNA (Fig. 2b). According to the GTEx portal, GS1-600G8-3 (here named *ATXN3L1-AS1*) is mainly expressed in testis, mirroring the expression pattern observed for the *ATXN3L1* transcript. Consistent with this, TSS activity mapped to the beginning of *ATXN3L1* 5′UTR (chrX:13,320,050-13,320,053; TTAT) was detected exclusively in adult testis. Both ReMap Atlas and ORegAnno databases indicate that this 5′UTR region is enriched for binding sites of multiple transcription factors. Additionally, a polyadenylation site within the *ATXN3L1* 3′UTR (chrX:13,318,643-13,318,669; ATTAAA@-21) was also used exclusively in testis, supporting tissue-specific transcriptional regulation (Fig. 2b).

*ATXN3L2* (ENSG00000253107) is also reported as a single exon with two predicted TSS (hg_90280.1 chr8:110555387-110555397; AGACAAATAAA; and hg_90281.1 chr8:110556281-110556292; AAGCAGCAGCAG) (Fig. 2c). Indeed, near the 5′ and 3′UTRs there are possible regulatory regions reported in ENCODE, ORegAnno and ReMap. However, there are no ESTs aligned to *ATXN3L2*, no polyA signal close to its 3′UTR, and no gene expression is reported in GTEx portal (Fig. 2c).

### Protein Conservation and Selective Tests

**Fig. 3.**
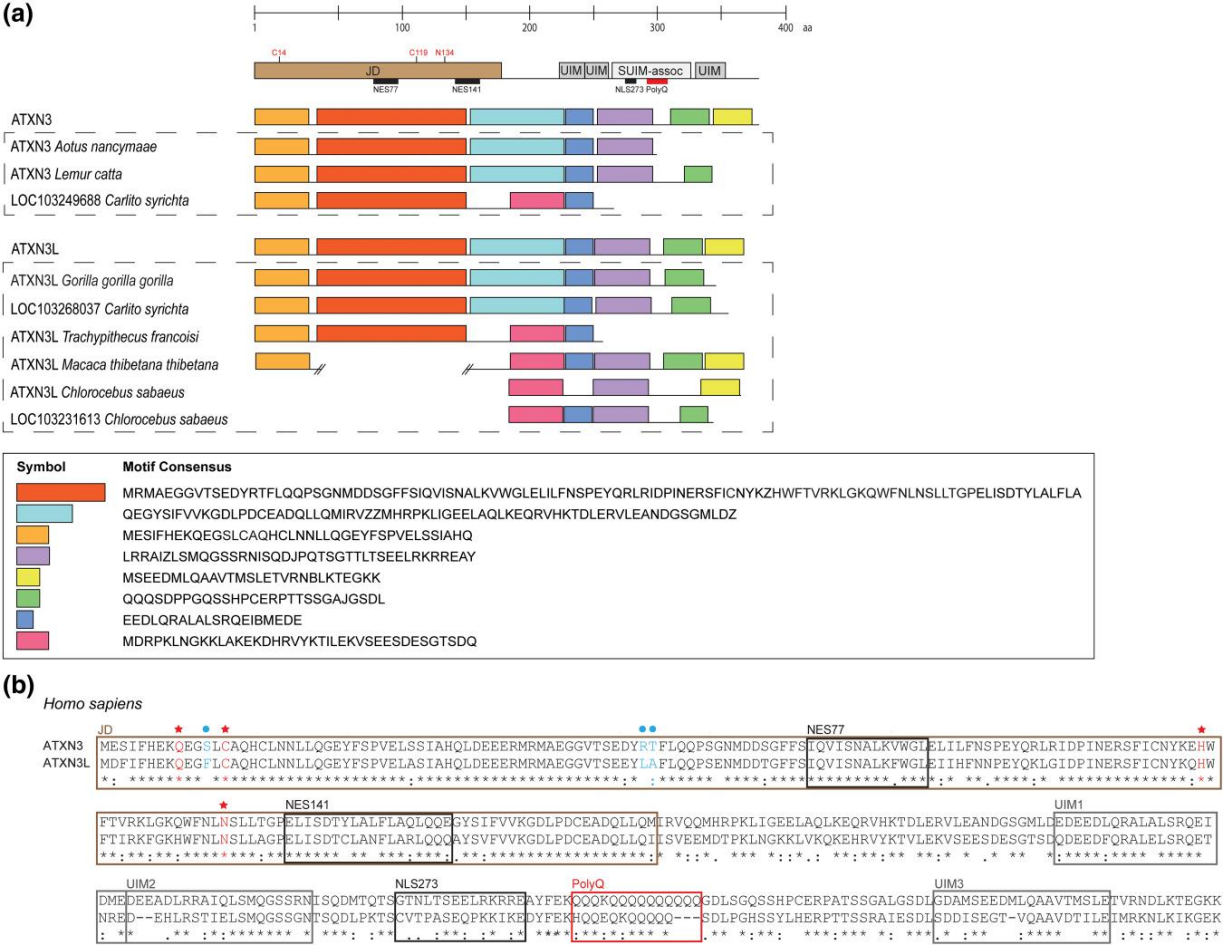
Conservation of ATXN3L1. a) Comparison of ATXN3 and ATXN3L1 conserved protein motifs (predicted in MEME suite) among primate species. b) Protein alignment of *Homo sapiens* ATXN3 and ATXN3L1 sequences, highlighting important domains, ATXN3 catalytic sites (Q9, C14, H119, N134—red stars), and three mutations (S12F, R59L, and T60A; called triple mutation—blue circles) found to almost match the ATXN3L1 and ATXN3 deubiquitinase activity. JD, Josephin domain. UIM, ubiquitin-interacting motif. SUIM-assoc, unstructured region C-term to UIM in ATXN3. NES, nuclear export signal. NLS, nuclear localization signal. PolyQ, polyglutamine stretch.

The predicted human ATXN3L1 protein sequence presents 70% of global identity with ATXN3 (Table S2b), preserves its catalytic sites (C14, H119, N134) and nuclear export signs (Fig. 3b) as well as the Josephin Domain (JD) at the N-terminal and three ubiquitin-interacting motifs (UIMs), at the C-terminal region (Tables S2c and S2d). Among haplorrhines, UIMs 1 to 3 show 83, 63% and 79% identity, respectively; however, unlike UIMs 1 and 2, the UIM3 is not always present, with both ATXN3 and ATXN3L1 missing this motif in a few primates due to alterations in the coding sequence (eg *Aotus nancymaae ATXN3* and *Trachypithecus francoisi ATXN3L1*; Fig. 3a; Fig. S1). Interestingly, in *Macaca thibethana thibetana* there seems to have occurred the insertion of an intron (Fig. 3a; Fig. S1) similar to one found in some *ATXN3* alternative splicing transcripts identified in humans (Bettencourt et al. 2010). On the other hand, the nuclear localization signal (NLS) sequence seems to be absent in ATXN3L1, raising the question of the subcellular location of this paralog.

**Fig. 4.**
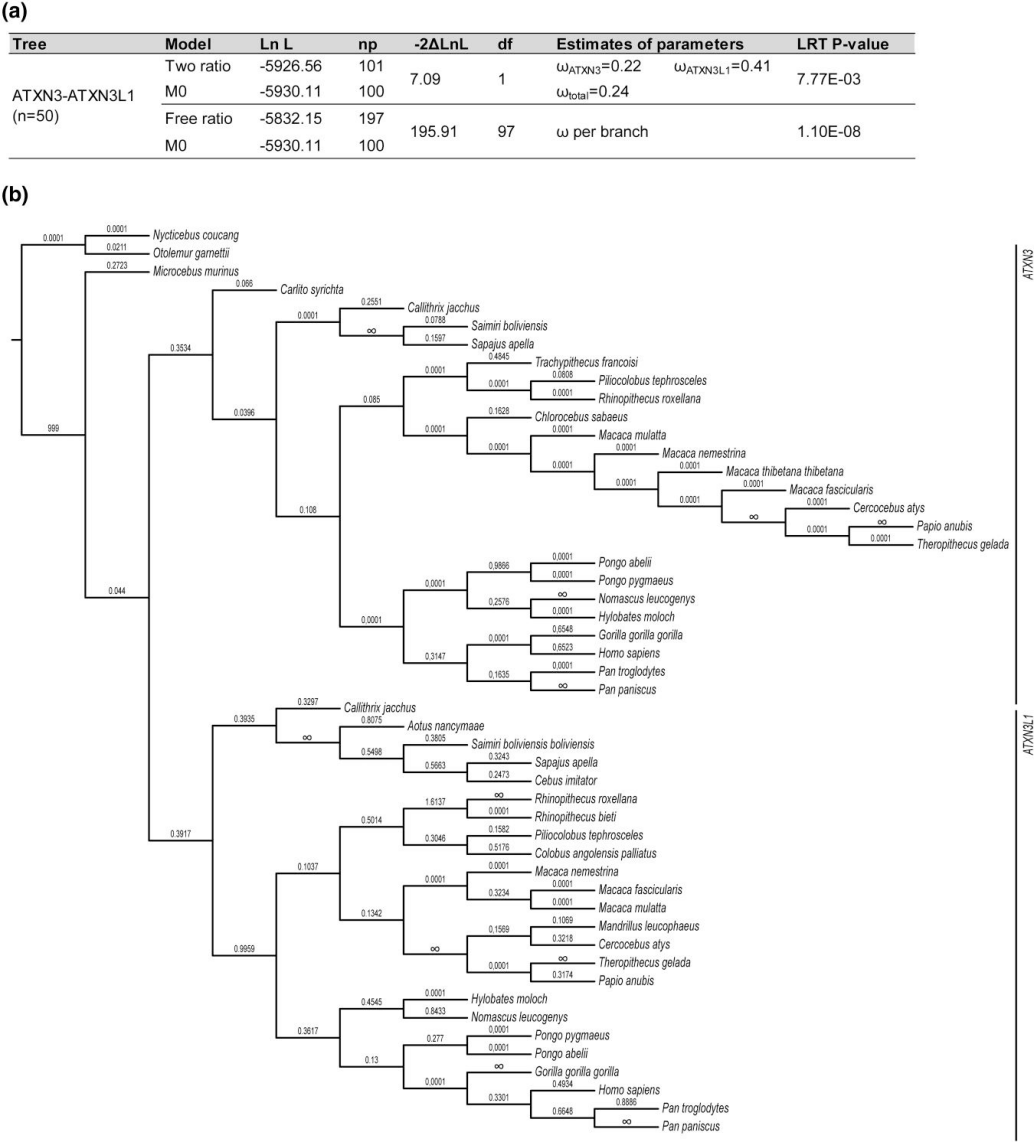
*ATXN3L1* shows an overall more permissive negative selection than the *ATXN3* gene clade. a) Model comparison of omega ratios calculated for the *ATXN3*-*ATXN3L1* tree—one-ratio (M0) versus two-ratio and free-ratio models. b) Cladogram of *ATXN3* and *ATXN3L1* gene lineages demonstrating PAML-estimated ω values under the free-ratio branch model, which assumes independent values on each branch. Values of infinity (∞) indicate branches lacking synonymous substitutions, and values of 0.0001 indicate branches lacking non-synonymous substitutions. LnL, log-likelihood. np, number of parameters. df, degrees of freedom. LRT, Likelihood ratio test.

To assess the intensity of the selective forces acting on both *ATXN3* and *ATXN3L1*, we performed several phylogenetic-based tests using *codeml* (PAML package), after removing the (CAG)_n_ region from sequence alignments. First, to evaluate the evolution of both genes in primates, we estimated a single ω ratio for the entire phylogeny (one-ratio model), in which we assumed no differentiation in selective constraints of *ATXN3* and *ATXN3L1* (ω_total_ = 0.23, Fig. 4a). Then, to examine whether the parental gene and retrocopy were subjected to different selective pressures, we applied a two-ratio model, which considers two branches within the phylogeny—the *ATXN3* or *ATXN3L1* clades. The parental gene is under stronger selective constraints (ω_ATXN3_ = 0.22) when compared to *ATXN3L1* (ω _ATXN3L1_ = 0.41, *P* = 7.77E−03; Fig. 4a). To investigate the strength of the selective constraints in *ATXN3L1*, we employed the RELAX model from the HyPhy software, which infers if relaxed or intensified selection has been acting along the selected branches by introducing a selection intensity parameter (k). Here, we validated that purifying selection has become less stringent along *ATXN3L1* branches in comparison with *ATXN3* (*k* = 0.00, LR = 60.79, *P* = 0.00E + 00).

Finally, we applied the free-ratio branch model to estimate a single omega value per each branch of the tree combining the two genes (Fig. 4b), which showed significant variation in ω values across the different branches (*ATXN3*-*ATXN3L1*, −2ΔLnL = 195.91; likelihood ratio test [LRT] *P*-values = 0.00; Fig. 4a). Overall, our results suggest strong selective constraints (negative selection) acting on *ATXN3* (ω = 0.044) prior to the event of retrotransposition, later followed by a period of a slight weakening in selective pressures driving the evolution of *ATXN3* (ω = 0.3534) and *ATXN3L1* (ω = 0.3917, Fig. 4b). Next, the selective forces were restored in *ATXN3* to the original strength in Tarsiiformes (ω = 0.066) and Simiformes (ω = 0.0396) ancestors, with several branches lacking non-synonymous substitutions (ω = 0.0001, Fig. 4b). For *ATXN3L1*, the selection was maintained as less restrictive but still compatible with functional conservation in both Platyrrhini (ω = 0.3935) and Catarrhini (ω = 0.9959) ancestors, with the existence of a period of accelerated evolution for the *Rhinopithecus* ancestor (ω = 1.6137, Fig. 4b).

**Fig. 5.**
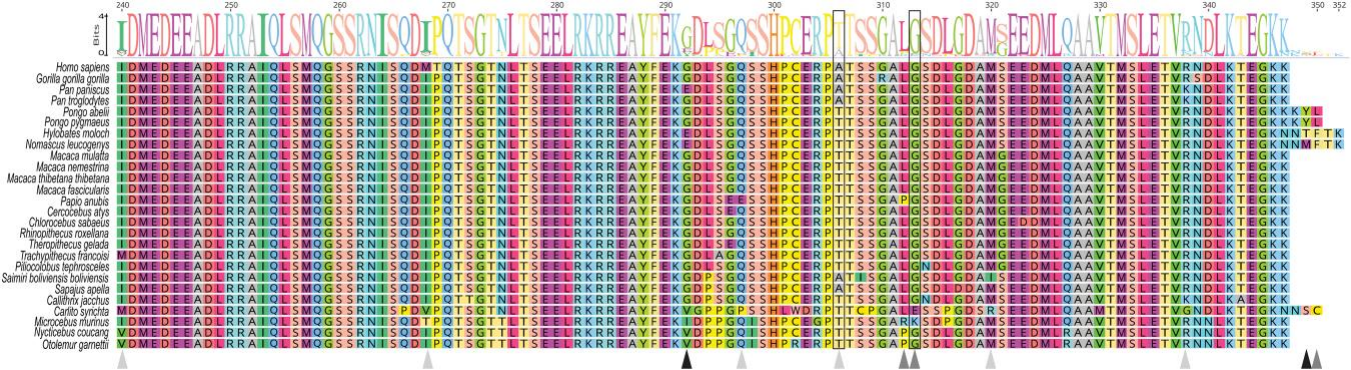
Positively selected sites in ATXN3. ATXN3 sites under positive selection identified by M8 site-model from PAML (pp > 0.5), and Fixed Effect Likelihood model (FEL; *P* ≤ 0.1) and the Mixed Effects Model of Evolution (MEME; *P* ≤ 0.05) from HyPhy/Datamonkey. Highlighted residues are colored according to PAML posterior probability scores (light gray: pp > 0.5, dark gray: pp > 0.7, black: pp > 0.9). Codons predicted by the two models are enclosed in a box. *Propithecus coquereli*, *Lemur catta*, *Colobus angolensis palliatus*, *Cebus imitator*, *Mandrillus leucophaeus*, *Aotus nancymaae* and *Rhinopithecus bieti* were not included in positive selection analyses due to sequences constraints.

Next, to identify regions in *ATXN3* and *ATXN3L1* that could have undergone adaptive evolution in primates, we performed *site-models* tests for each gene separately. According to our results, only the parental gene shows some evidence of diversifying evolution in some codons (Table 3: M1a vs. M2a; M7 vs. M8; Fig. 5), with less than 1% of ATXN3 sites showing high omega values (M8 vs. M7 p1 = 2.95E−03, ω = 297.94). Indeed, only 11 codons have a posterior probability (pp) > 0.5 for the positive-selection class (Table 3). When considering merely the sites with a pp > 70%, just five codons have a high probability of being positively selected: 292, 312, 313, 349, and 350. Three of them are located between UIM2 and UIM3 domains, in a disordered region flanking the polyQ repeat (Fig. 3b and 5). Codon 292 encodes the first residue downstream the polyQ, and codons 312 and 313 are close to the start of ATXN3 UIM3. Codons 349 and 350 encode additional C-terminal residues specific to orangutan (*Pongo pygmaeus*, *Pongo abelii*), gibbons (*Hylobates moloch*, *Nomascus leucogenys*), and tarsier (*Carlito syrichta*) (Fig. 5). In fact, these species have alterations at the end of exon 11, resulting in the extension of the coding sequence (Fig. S1), which makes these two C-terminal amino acids unlikely to have a great functional impact.

**Table 3:** Parameter estimates and likelihood scores under different branch and site models

Tree	Model	Ln L	np	Model comparison	−2ΔLnL	df	Estimates of parameters				LRT *P*-value	Positive sites
ATXN3 (*n* = 26)	M3	−2919.22	55	M0 versus M3	79.24	4	*P*:	0.89	0.11	0.00	0.00E+00	…
							ω:	0.03	0.78	309.75		…
	M0	−2958.84	51				ω0:	0.10				…
	M2a	−2919.50	54	M1a versus M2a	21.35	2	*P*:	0.91	0.09	0.00	2.31E−05	…
							ω:	0.04	1.00	317.32		…
	M1a	−2930.17	52				*P*:	0.91	0.09	…		…
							ω:	0.04	1.00	…		…
	M8	−2922.21	54	M7 versusM8	25.17	2	p0 = 1.00	*P* = 0.01	q = 0.03	…	3.42E−06	240 I 0.560, 268 M 0.623, 292 G 0.921, 297 Q 0.570, 306 A 0.649, 312 L 0.815, 313 G 0.720, 320 M 0.503, 338 R 0.553, 349–0.985, 350–0.785
							(p1 = 2.95E−03)	ω= 297.94	…	…		
	M7	−2934.79	52				*P*=	0.02	q=	0.11		…
	M8a	−2930.12	53	M8a versusM8	15.83	1	p0 = 0.92	*P* = 0.57	q = 10.79		6.93E−05	…
							(p1 = 0.08)	ω= 1.00				…
ATXN3L1 (*n* = 24)	M3	−3735.68	51	M0 versus M3	36.65	4	*P*:	0.57	0.41	0.01	2.13E−07	…
							ω:	0.13	0.71	3.43		…
	M0	−3754.01	47				ω0:	0.37				…
	M2a	−3736.13	50	M1a versus M2a	2.23	2	*P*:	0.73	0.26	0.01	3.28E−01	…
							ω:	0.19	1.00	3.85		…
	M1a	−3737.24	48				*P*:	0.72	0.28	…		…
							ω:	0.18	1.00	…		…
	M8	−3735.82	50	M7 versusM8	5.23	2	p0 = 0.98	*P* = 0.64	q = 1.07	…	7.31E−02	…
							(p1 = 0.02)	ω= 3.32	…	…		…
	M7	−3738.43	48				*P*=	0.475	q=	0.71		…
	M8a	−3737.24	49	M8a versusM8	2.85	1	p0 = 0.72	*P* = 21.48	q = 99.00		9.14E−02	…
							(p1 = 0.27)	ω= 1.00				…

LnL, log-likelihood; np, number of parameters; df, degree of freedom; LRT, Likelihood ratio test.

For ATXN3L1, no codons were identified as experiencing positive selection (M1a vs. M2a: 2ΔLnL = 2.23, *P* = 3.28E−01; M7 vs. M8: 2ΔLnL = 5.23, *P* = 7.31E−02; M8 vs. M8a: 2ΔLnL = 2.85, *P* = 9.14E−02).

To corroborate PAML results for *ATXN3*, we additionally used two models implemented through HyPhy/Datamonkey: Fixed Effect Likelihood model (FEL, with *P* ≤ 0.1) and Mixed Effects Model of Evolution (MEME, with *P* ≤ 0.05). The first model assumes a constant selective pressure for each site, while the second presume that selective pressures between branches are uncorrelated or independent. FEL model identified 93 codons under purifying selection distributed along the ATXN3 protein, mostly in the N-terminal that includes the Josephin domain and nuclear export signs (from codon 1 to 180; Table S3a). Interestingly, we found codon 313 to be undergoing diversifying selection throughout the phylogeny (β = 1.652, LRT = 2.843, *P* = 0.09) (Table S3a), as predicted by PAML (pp = 0.720). In parallel, the MEME model showed codon 306 (LRT = 6.80, *P* = 0.01) as an episodic positive selected site (Table S3b), also predicted in PAML site model (pp = 0.649). When accounting for the results from both PAML and HyPhy, we observed that the positively selected sites are in the C-terminal region of ATXN3, which includes UIM3 (Fig. 5).

## Discussion

In this study, we identified four main retrotransposition events of *ATXN3*. Besides the previously reported *ATXN3L1*, we identified an unannotated pseudogene probably originated in Euarchontoglires (here named as *ATXN3L0*) and two not fully characterized genes (herein called *ATXN3L2* and *ATXN3L3*) that arose in Simiformes and Cercopithecidae ancestors, respectively (Table 1, Fig. 1b). Additional retrotransposition events occurred more recently, either in a single species or in a restricted clade (Table S1). These findings are not surprising given several studies suggesting a high rate of retrotransposition in mammals, especially in primates, probably driven by their enrichment in full-length LINE 1 elements (Ivancevic et al. 2016). Studies pointed to periods of intense activity of transposons around the mammalian radiation, in Simiiformes (40.0 to 44.2 MYA), Haplorrhini (61.6 to 71.1 MYA), and Eutheria (placental mammals, 94.7 to 101.9 MYA) ancestors (Marques et al. 2005; Pace and Feschotte 2007), in concordance with age estimates of *ATXN3* retrotransposition. Also, during the search for *ATXN3L0* copies in Mammalia species, no retrocopies were found in Prototheria, in concordance with the previously report that genomes from Monotremata species have no LINE1 elements (Ivancevic et al. 2016). On the other side, in disagreement with the lack of evidence for the activity of transposons after the emergence of Cercopithecidae (with no LINE1 younger than 37 million years; Pace and Feschotte 2007), we identified recent retrotransposition events, which may indicate that a few active elements were able to be transmitted within these primates.

The identification of sequences likely to be homologous to *ATXN3L0* in some non-primate mammals though absent among rodents (with the exception of the Sciuridae family) could mean that (i) a large-scale loss of *ATXN3L0* occurred in rodents related species, (ii) rapid evolution of *ATXN3L0* made this retrocopy no longer identifiable in some species from Supraprimates, or (iii) some independent retrotransposition events of *ATXN3* occurred in some species resulting in similar homologs when compared with *ATXN3L0*.

*ATXN3L1* was first thought to have originated in a Catarrhini ancestor (Weeks et al. 2011), and later in the Simiiformes infraorder, after the separation of the Tarsiiformes lineage (Sousa e Silva et al. 2023); however, in this study, we established the time of origin of *ATXN3L1* to a Haplorrhini ancestor (Fig. 1b), approximately 25.7 million years earlier. The absence of *ATXN3L1* tandem copies in some Old-World monkeys, as well as *ATXN3L0* and *ATXN3L2* retrocopies in a few species is possibly due to gene loss events after speciation (Table 1 and Fig. 1b), although the possibility of incomplete genomic data cannot be excluded. Besides, we should also consider that gene losses may be a species-specific event, not occurred in other species from the same order or family. Another difficulty we faced on the identification of paralogs/orthologs were discrepancies in synteny, which may be expected once chromosomal rearrangements are known to have occurred after speciation (Chiou et al. 2022), as seen in *Theropithecus gelada*, as we found synteny blocks from chr. X differently positioned in comparison to *Papio anubis* (Fig. 1a and Table S1).

Interestingly, *ATXN3L1* was retrotransposed from an autosome to the X chromosome, contrarily to most reports of X-linked genes being transposed to autosomal chromosomes, with mammalian X chromosomes producing a disproportionately high number of functional retroposed genes in comparison to autosomes (Emerson et al. 2004; Kaessmann 2010). Still, *ATXN3L1* seems to exhibit the same testis-biased expression as previously described for X-linked retrotransposed genes. Previous studies have discussed the role of testis in the process of gene evolution, allowing young paralogs to evolve, fixate, and later broaden their expression pattern in somatic tissues with time (Kaessmann 2010; Guschanski et al. 2017). Additionally, studies have been reporting an intrinsic relation between testis and brain expression (Guo et al. 2003), where these tissues share numerous common proteins, evidencing mutual biochemical and functional features (Matos et al. 2021). This way, we cannot discard the hypothesis of *ATXN3L1* being expressed in other tissues given the lack of functional studies involving this retrogene, including a precise quantitative transcript expression analysis. For instance, if this retrogene is expressed in specific structures or cell types of the adult brain, or even during stages of brain development (not previously observed), this paralog could gain further relevance in the study of Machado–Joseph disease. It is certain, however, that *ATXN3L1* is a transcriptionally active gene, which either retained the *ATXN3* promoter during the retrotransposition event (Okamura and Nakai 2008) or acquired a nearby promoter element (the 5′ closest gene to *ATXN3L1* is *EGFL6*; enriched in placenta, according to GTEx). In humans, *ATXN3L1* retrogene is located within an antisense long non-coding RNAs (lncRNAs) missing annotation and functional characterization (Fig. 2b). It is known that, in the human genome, some retrocopies overlap with lncRNAs, regulatory RNA sequences of >200 nucleotides that can establish specific interactions with RNA molecules and proteins involved in the expression of the parental gene (Bryzghalov et al. 2016). Therefore, it would be crucial to characterize the targets of this unknown lncRNA gene and its effect on RNA expression, especially on sequences highly similar to *ATXN3*.

Additionally, the fact that *ATXN3L1* is located on the X chromosome may be interesting to explore gene expression dosage and how this could potentially be relevant to MJD pathogenesis. Mammals have evolved a compensatory mechanism to randomly inactivate one of the female X chromosomes in somatic cells, however, a number of genes located at the p-arm escape X inactivation (15% to 25% of all known ChrX genes; Balaton et al. 2015). These genes are called XCI escape genes or escapees and, in humans, the vast majority is located on the short arm-Xp, as *ATXN3L1* (Xp22.2) (Disteche 1999). Although no differences in MJD phenotype are known between male and female patients (Coutinho 1992), it would be interesting to explore this possibility.

Both *ATXN3* and *ATXN3L1* seem to be under purifying selection, with the retrocopy experiencing less selective constraints than the parental gene (Fig. 4a). This is not surprising since most genes involved in severe monogenic diseases are reported to be under strong negative selection (Blekhman et al. 2008). Besides, duplicates of genes with high biological relevance have often been observed under more relaxing constraints, thus promoting functional divergence (eg subfunctionalization) (Jordan et al. 2004); still, a retained purifying selection allows the maintenance of functional utility, in accordance with our results for *ATXN3L1* (Fig. 4b). Interestingly, previous studies have shown that the orthologs evolutionary rates for duplicates have been negatively correlated with the number of paralogs and the strength of selection between paralogs (Jordan et al. 2004); this may indicate that the number of *ATXN3* paralogs is leading to an overall conservation of all copies.

The hypothesis of *ATXN3L1* being conserved and functionally relevant is reinforced by its high similarity with *ATXN3*: the Josephin domain is putatively maintained, as well as the two essential ubiquitin-interacting motifs (UIM1 and UIM2, the only ones present in the shorter isoform of human ATXN3), and the potential nuclear export signal (Fig. 3). Although there are no studies on the subcellular localization or molecular targets of ATXN3L1, the conservation of the catalytic triad (C14, H119, N134) and nuclear export signs when compared with ATXN3 is a good indication of its deubiquitinase function in the cytoplasm. Indeed, the ATXN3L1 Josephin domain adopts the same overall fold as the ataxin-3 Josephin domain, and in vitro assays demonstrated its catalytic activity (Weeks et al. 2011), which is not surprising given the 85% sequence identity between the two domains. At the same time, we cannot discard the hypothesis of new functions being ascribed to ATXN3L1, which would be of upmost importance to help clarifying the biological functions of wild-type (non-expanded) ATXN3, still unknown.

Intriguingly, despite the similarities between ATXN3 and ATXN3L1, only the parental protein seems to contain some fast-evolving residues. Those are all located in the C-terminal region of ATXN3, which, according with the three-dimensional model includes a disordered region, followed by the polyQ and the UIM3 (Fig. 5). These ATXN3 residues can be under diversifying selective pressures that alter protein properties related to the ubiquitin interaction, or the region around UIM3 may not be critical for its activity and thus not subjected to negative constraints. Indeed, shorter *ATXN3* protein-coding transcripts that include only UIM1 and UIM2 have been shown to be more abundant in the brain, while UIM3-containing transcripts were more frequent in blood (Raposo et al. 2024). Therefore, this pattern of selective pressures in *ATXN3* may be linked to Machado–Joseph disease pathogenesis.

*ATXN3L2* presents a high nucleotide sequence conservation when compared with *ATXN3* (Table S2a), but multiple frameshift mutations and premature stop codons during primate evolution may have turned it into a non-functional processed pseudogene. This does not prevent, however, the transcription of *ATXN3L2* (Fig. 2c), which may play a role in expression regulation of the parental gene. In fact, in SCA7, it was previously proposed that the retropseudogene *ATXN7L3B* (which accumulated frameshift mutations since its duplication, resulting in premature stop codons and a truncated open reading frames [ORF]) acts as a post-transcriptional regulator of *ATXN7*, contributing to specific neurodegeneration (Tan et al. 2014). Also, previous studies have demonstrated that processed pseudogenes are capable of regulating the expression of parental genes through different mechanisms such as (i) antisense transcripts forming stable RNA duplexes with the parental mRNAs inhibiting the translation, (ii) long noncoding RNAs (lncRNAs) acting as molecular sponges for RNA-binding proteins (RBPs) or microRNAs through shared binding sites, and (iii) small-interfering RNA (siRNAs) acting as a decoy for transcription factors (Cheetham et al. 2020).

Finally, the analysis of *ATXN3* repeat sequences in other non-human primates and paralogs provided some insight into the mutational mechanisms that underlies the expansion of the CAG repeat associated with MJD in humans. The observation that only certain repeat sequences become unstable was suggested to be related to sequence-specific DNA structures, potentially crucial for instability. In *ATXN3*, gene regions surrounding the (CAG)_n_ were shown to be highly conserved in primates, whereas the mutation rate at the two codons adjacent to the repeat was estimated to be 27 times higher than in intronic regions (Djian et al. 1996). The fact that *ATXN3L1* exhibits more interrupted and stable (CAG)_n_ configurations over primate evolution suggests that the ancestral (CAG)_n_ region of *ATXN3* was highly interrupted. However, mutations in *ATXN3* over the last 61.6 to 71.1 MYA likely led to a less interrupted (CAG)_n_ region, increasing its susceptibility for expansion. In addition to the analysis of configuration patterns, it would be interesting to study the polymorphic nature of repetitive regions in all retrocopies in several primates.

In this study, we reconstructed the history of *ATXN3* gene family and performed a comprehensive evolutionary analysis to gain insight into the functional relevance of *ATXN3* paralogs. *ATXN3* was described three decades ago, and it has been extensively explored due to its implication in Machado–Joseph disease (MJD/SCA3), the most frequent dominant ataxia worldwide. Interestingly, increased retrotransposition activity has been suggested to be linked to neurodegenerative disorders (Terry and Devine 2020). This may be related to the fact that brain is the only known somatic tissue where retrotransposons are de-repressed in humans, whereas, in other tissues, silencing mechanisms restrict retrotransposition due to its inherited capacity to disrupt genomes and transcriptomes (Yang and Wang 2016). Several studies indicate that retrotransposed genes may be important players in pathogenesis by influencing transcriptional and post-transcriptional expression of disease-related genes (Ciomborowska-basheer et al. 2021). Our study highlights the value of evolutionary analyses to gain insight into the potential functional relevance of unexplored duplicates. This approach enables a more effectively identification of the most promising candidates for subsequent in vitro assays and experimental testing of their roles as disease modifiers.

## Materials and Methods

### Identification of *ATXN3* Homologous Genes and Orthology/Paralogy Inference in Primate Species

We conducted tblastn searches against 33 representative and RefSeq annotated primate assemblies (Table S4; Supplemental files S1 and S2) in the Basic Local Alignment Search Tool (BLAST, https://blast.ncbi.nlm.nih.gov/Blast.cgi; accessed July 28, 2023) (Camacho et al. 2009) from National Center for Biotechnology Information (NCBI) using *Homo sapiens* reference *ATXN3* protein sequence (NP_004984.2) as input. To identify genes that might be missing from previous studies and databases, we inferred orthology and paralogy through local synteny analysis in Genome Data Viewer (https://www.ncbi.nlm.nih.gov/genome/gdv/) from NCBI.

### Search for *ATXN3L0* Orthologs in Mammalian Species

To ascertain the origin of *ATXN3L0* retrotransposition event, we performed tblastn searches on other 58 representative genomes of mammals (accessed October 18, 2023; Table S4) using the predicted protein sequence obtained for *Homo sapiens* (LOC100418768). The focus was on species from the Euarchontoglires superorder.

### Collection of Nucleotide and Protein Sequences

The sequences of the genes identified in the tblastn analysis, together with their annotation data, were directly retrieved from NCBI's GenBank nucleotide database. In addition, homologous sequences from *Gallus gallus* and *Anas platyrhynchos* were used as outgroup in the for the complete nucleotide alignment with all paralogs.

Paralogs and orthologs sequences were aligned using MUSCLE implemented in Geneious Prime software (2022.2.2, www.geneious.com) and manually curated/inspected according with sequence conservation among species.

We used Augustus gene prediction algorithm implemented in Geneious Prime to predict ORF and potential coding regions. We obtained protein sequences from the translation of collected nucleotide sequences and calculated nucleotide and protein identity percentage (%) matrices in Geneious Prime.

### Phylogenetic Analysis

To determine the best nucleotide substitution model for tree estimation, we used jModelTest version 2.1.10 (Darriba et al. 2012). For *ATXN3*, *ATXN3L1*, and *ATXN3*-*ATXN3L1* (phylip files, without CAG regions), we identified the TIM1 + G (transition model with proportion of invariable site and rate heterogeneity across sites; code 012230), GTR + G (general time reversible model with rate heterogeneity across sites), and GTR + I + G (general time reversible model with proportion of invariable sites and rate heterogeneity across sites) models, respectively, as the most suitable ones. When considering the alignment with all nucleotide sequences and the (CAG)_n_ region, the TVM + I + G (transversion model with proportion of invariable sites and rate heterogeneity across sites; code 012314) model was found to be the best-fitting model.

We inferred the maximum likelihood tree using the PhyML algorithm implemented in Geneious Prime under default parameters (analyses were performed with 1,000 bootstrap searches); and next displayed the phylogenetic trees using FigTree v1.4.4 (http://tree.bio.ed.ac.uk/software/figtree/).

### Selective Pressure Analysis in *ATXN3* and *ATXN3L1*

To evaluate the selective pressures acting on *ATXN3* and *ATXN3L1*, we employed the *codeml* program from Phylogenetic Analysis by Maximum Likelihood (PAML) (Yang 2007) package using the EasyCodeML tool (Gao and Ho 2019). Sequences were only included after the removal of positions affected by premature stop codons and frameshift mutations. To test for variable selective pressures among branches, we performed the branch model using either the null model (one ratio) or the nested model (two-ratio) (Bielawski and Yang 2003). We calculated the ratio of nonsynonymous (dN) to synonymous (dS) substitution rates (ω, omega), assuming distinct neutral or selective models, with ω = 1, >1 or <1 expected in a neutral, positive, or purifying (negative) evolution, respectively. Models implemented in *codeml* vary in terms of their assumptions about ω variation across the sequence or across branches of the phylogeny. Firstly, we used branch models (one-ratio vs. free-ratio, one-ratio vs. two-ratio) to test the hypothesis of equal evolutionary rates between *ATXN3* (background) and *ATXN3L1* (foreground) gene lineages after retrotransposition. The Free-ratio model allowed to analyze selection constraints in all species for both *ATXN3* and *ATXN3L1* genes. Next, we used site-specific (M0 vs. M3, M1a vs. M2a, M7 vs. M8, M8 vs. M8a) models to test if certain codons of *ATXN3* and *ATXN3L1* were under positive selection in haplorrhines. For branch models each newick (.nwk) tree was altered to label the clade of interest as foreground (inserted with $) and the remaining branches as background.

We used LRTs to perform pairwise comparisons between codon models and obtained significant results from twice the variation of likelihoods (−2Δl) using χ^2^ tests. If LRT yielded a significant result for M7 versus M8, the Bayes empirical Bayes procedure was used to identify codons/amino acids under positive selection (posterior probability ≥ 0.5).

Additionally, we used the Hypothesis Testing using Phylogenies (Hyphy) software implemented in the Datamonkey Adaptive Evolution Server (https://www.datamonkey.org/) (Weaver et al. 2018) to detect sites under selection in codon-based alignments with the Fixed Effect Likelihood model (FEL) and the Mixed Effects Model of Evolution (MEME) (Kosakovsky Pond and Frost 2005; Anisimova 2012; Murrell et al. 2012). The FEL model is based on a maximum-likelihood approach to infer dN and dS substitution rates, assuming that the selection pressure for each site is constant along the phylogeny. The MEME model employs a mixed-effects maximum likelihood approach where the ω on each branch is independent of those from other branches, ie selective pressures between branches are uncorrelated. This way, each site has two ω rate classes and corresponding weights representing the probability of the site to evolve under ω rate class, at a given branch. To infer ω rates, MEME infers a single α (dS) value and two separate β (dN) values, β− and β+, which share the same α, per site. In short, MEME model allows ω to vary between sites and branches, detecting both episodic and pervasive positive selection. We also used LRTs to ascertain significant results (*P* ≤ 0.1 and *P* ≤ 0.05, respectively). Next, test whether the strength of natural selection has been relaxed or intensified along the *ATXN3L1* clade in comparison with *ATXN3*, we used the RELAX model (Wertheim et al. 2015). After fitting a codon model with three ω parameters (ω0 ≤ω1 ≤ 1 ≤ω2) to the entire phylogeny (null model), the RELAX model determined the proportion of sites in *ATXN3L1* (foreground) and *ATXN3* (background) branches using a branch-site model. Additionally, a selection intensity parameter (*k*) was introduced to compare a null model (*k* = 1) with an alternative model, assessing if the strength of natural selection has been intensified (*k* > 1) or relaxed (*k* < 1) in the *ATXN3L1* branch relative to the *ATXN3* branch. Statistical confidence (*P* ≤ 0.05) was calculated with LRTs and the Holm-Bonferroni correction.

### Identification of *ATXN3L1* Conserved Motifs

To identify conserved motifs possibly essential for gene function, we used the Multiple EM (Expectation Maximization) for Motif Elicitation (MEME) tool (https://meme-suite.org/meme/tools/meme, version 5.5.4) from MEME Suite (Bailey and Elkan 1994; Bailey et al. 2009). Parameters adjusted for MEME analysis included: number of repetitions (zero or one occurrence per sequence (zoops)), maximum number of motifs (15, ordered by E-value) and width of sequence (20 to 200 amino acids). We used the ATXN3-ATXN3L1 protein fasta file aligned and concatenated as input to analyze conserved regions likely to be contributing to protein structure and function.

### Genomic Characterization of *ATXN3* Human Paralogs

We performed the genomic characterization of *ATXN3* human paralogs (*ATXN3L0*, *ATXN3L1,* and *ATXN3L2*) through the UCSC Genome Browser (https://genome.ucsc.edu/; Nassar et al. 2023) displaying summarized regulatory and chromatin-related features from ENCODE project (cis-regulatory elements, DNase I hypersensitivity peaks, and H3K4Me1/H3K4Me3/H3K27Ac histone marks), and ORegAnno database (predicted regulatory elements). Also, we assessed predicted promoters (EPDnew v006), transcription start sites (TSS; FANTOM5), and poly(A) sites (custom track from PolyASite v.2.0; https://www.polyasite.unibas.ch/; Herrmann et al. 2020). Along these transcriptional indicators, we used human mRNAs from GenBank to verify the previous identified RNAs of these paralogs.

## Supplementary Material

evag047_Supplementary_Data

## Data Availability

All datasets were derived from sources in the public domain. The data and analysis underlying this article are available in the article and in its online Supplementary material.
